# Evaluating the Role of Compression Stockings in Preventing Post thrombotic Syndrome: A Review of the Literature

**DOI:** 10.1155/2012/694851

**Published:** 2012-01-17

**Authors:** Abir O. Kanaan, Jayne E. Lepage, Shabdis Djazayeri, Jennifer L. Donovan

**Affiliations:** Department of Pharmacy Practice, College of Pharmacy, Massachusetts College of Pharmacy and Health Sciences, 19 Foster Street, Worcester, MA 01608, USA

## Abstract

*Background*. Postthrombotic syndrome (PTS) is a burdensome and costly complication of deep vein thrombosis (DVT). Up to 50% of patients with DVT will develop the disease within two years following the diagnosis of acute DVT. Various risk factors for developing PTS have been identified and different modalities have been used to prevent its development. Compression stockings have been studied for the prevention of PTS in patients diagnosed with proximal DVT. *Methods*. MEDLINE and EMBASE databases were searched to identify relevant original articles. *Results*. Several trials including two metaanalyses have examined the role of compression stockings for the prevention of PTS. Although most trials showed significant reduction in the development of PTS with the use of compression stockings, limitations in study design prevent the generalizability of the data. Two studies supported an individualized tailored duration especially in patients at low risk for developing the syndrome. A randomized double-blind placebo-controlled trial involving 800 patients is currently ongoing and may confirm the results of older studies. *Conclusions*. Clinical trials support the use of compression stockings in patients diagnosed with proximal DVT for the prevention of PTS.

## 1. Introduction

Postthrombotic syndrome (PTS) is a severe and common chronic complication of deep vein thrombosis (DVT) of the lower extremity. Between 20% and 50% of patients diagnosed with and adequately treated for a DVT will be affected with PTS within the following two- to five-years; with most cases being reported within the first two years [1–7]. However, the exact incidence and prevalence of PTS remain elusive due to inconsistency in the diagnosis of PTS, underreporting of most DVTs, and the lack of standardization of the definition of PTS [8]. In a population-based study published in 2000, the cumulative rates of PTS following the diagnosis of DVT ranged from 7% at 1 year to 27% at 20 years [9]. However, as the number of patients diagnosed with DVT is increasing in the United States and reaching 250,000 new cases annually, the prevalence and thus incidence of PTS will likely follow suit [10].

Classical features of PTS range from mild to severe and are difficult to quantify. The Villalta score combines signs and symptoms in a graded system and is recognized as the best tool in defining PTS [8]. Signs and symptoms of PTS include aching pain while standing, burst of pain while walking, edema, and itching of the affected limb [11, 12]. In severe PTS, affecting 10% of patients, intractable limb ulceration is common and is often a result of trauma [12]. The constellation of these symptoms leads to restricted mobility, limited daily activities, and adversely affects quality of life [13–17]. In fact, it is estimated that 2 million workdays are lost in the United States every year due to leg ulcers [18].

Healthcare costs for treating PTS vary among studies [16–22]. Some report that the mean incremental adjusted healthcare cost of developing PTS was approximately $7000/patient/year whereas others report a much lower rate of $400–$1200/patient/year [21, 22]. Nonetheless, there is agreement that the costs are significant and so, treatment is often ineffective that perhaps a better strategy may be to focus on the prevention of PTS. Therefore, the purpose of this article is to describe the available literature on the use of compression stockings in preventing PTS in adult patients.

## 2. Pathophysiology

The pathophysiology of PTS is not well defined. It is postulated that PTS occurs as a result of chronic venous hypertension that develops due to valvular reflex and residual thrombus. When a patient develops a DVT, damage to venous valves ensues as a result of acute thrombus, the process of vein recanalization, release of inflammatory mediators, fibrous scaring, or venous dilation with increased tissue permeability. These factors alone or in combination lead to valvular incompetence or reflux [23–26]. Moreover, treatment of a DVT with various anticoagulants prevents the extension and embolization of the thrombus rather than enhancing its clearance through disintegration. This leads to persistent venous obstruction due to residual thrombus. Of the two mechanisms leading to venous hypertension, the presence of residual thrombus alone or in combination with valvular reflux predicts the development of PTS more than valvular reflux alone. Thus, persistent venous obstruction due to the presence of residual thrombus appears to be a more important factor in the development of PTS [27–31].

## 3. Diagnostic Tools

The diagnosis of PTS is based on clinical findings in patients with previously confirmed diagnosis of DVT of the affected limb. These findings represent a collection of nonspecific signs and symptoms with a high degree of interpatient variability resulting in the classification of PTS as a syndrome. In some patients, the pain and swelling associated with the initial DVT making take up to 6 months to resolve and thus the diagnosis of PTS should be deferred to after the acute phase of DVT has elapsed to allow time for after revascularization symptoms of the initial DVT to resolve [1, 32].

Patients with PTS usually experience limb heaviness or fatigue, chronic pain, itching, cramping, and numbness that are worse with standing or activity and relieved with rest and elevation. Symptoms are typically present with varying frequency and intensity and are patient-specific. Physical examination of the limb may reveal edema, telangiactasias, hyperpigmentation, venous eczema, secondary varicose veins, and lipodermatosclerosis. In severe cases, leg ulcers requiring medical attention may be observed [1, 33] (Table 1).

Invasive and noninvasive diagnostic tools can be used to confirm the diagnosis of PTS especially in patients who present with symptoms of the syndrome but do not have a confirmed diagnosis of DVT. These tools should not, however, be utilized in the absence of clinical features for PTS as many patients with DVT will have abnormal findings similar to those presenting with PTS. Thus, the diagnosis of PTS is based on clinical presentation irrespective of the abnormalities shown by these diagnostic tools [43–45]. Contrast venography is an invasive, expensive tool used to identify findings that suggest a previous DVT. Compression ultrasonography along with continuous-wave Doppler scan are noninvasive measures that evaluate the compressibility of common femoral and popliteal veins and the reflux of venous valves. These diagnostic tests are especially helpful in patients who have features of PTS but without a history of DVT. Computed tomography venography and magnetic resonance venography can delineate thrombotic obstruction to plan for interventions. Three clinical scales utilize combinations of clinical signs and imaging to assess the presence and/or severity of PTS. These scales all recognize some combination of subjective signs and symptoms in patients with DVT after revascularization and vary in their consideration and weighting of objective evidence via plethysmography and/or venous Doppler and their incorporation of rating scales of significance of signs and symptoms. The Villalta scale combines patient limb symptoms and signs in a graded scoring system; the higher the score, the greater the severity of PTS. The Clinical-Etiology-Anatomic-Pathophysiologic (CEAP) scale has parameters that include clinical findings and subcategories to designate etiology, anatomic distribution, and pathophysiology [34–37, 40, 46] (Table 2). In an attempt to standardize the definition and measurement of PTS, the Subcommittee on Control of Anticoagulation of the Scientific and Standardization Committee of the International Society on Thrombosis and Haemostasis recommended the Villalta scale be adopted to diagnose and grade PTS in clinical research [8].

## 4. Risk Factors for PTS

A variety of clinical and patient-specific risk factors have been implicated in the development of PTS. These will be categorized into those that are evident at the time of diagnosis or during initial treatment of DVT, and those that develop in the long-term period following the management of DVT.

## 5. Risk Factors Evident at the Time or during Initial Treatment of DVT

### 5.1. Clinical Features

A definitive correlation between PTS and the type of DVT, whether it is idiopathic or provoked (cancer-related or modifiable risk factors), has not been established in prospective studies [7, 47, 48]. Moreover, data from various studies regarding the association between the development of PTS and location of the initial DVT have been inconclusive due to differences in study design, patient selection, and PTS definition. For instance, in some studies, patients presenting with proximal DVT were reported to have a higher incidence of developing PTS compared to those presenting with distal (calf) DVT [48–51]. In a prospective cohort study, patients with more extensive proximal DVT and increased clinical findings at 1 month were at an increased risk for developing PTS 2 years following DVT diagnosis. This could be attributed to persistence of residual thrombus [5]. However, literature from other studies reported up to 80% incidence of PTS in patients presenting with distal (calf) DVT; thus suggesting that this type of DVT may be associated with a considerable risk of PTS development [52, 53]. Moreover, pulmonary embolism without concomitant ultrasonographically detectable DVT does not predispose patients to PTS [54].

Finally, patients who develop DVT but are asymptomatic have an overall relative risk of 1.6 (95% CI 1.24–2.02) compared to patients without DVT [55]. In a prospective study, 20% of asymptomatic patients diagnosed with DVT following screening after total knee and total hip arthroplasty developed PTS by the 18-month followup period [56]. In a retrospective review of 1037 patients who underwent total hip arthroplasty, 21 patients developed DVT on postoperative day 3 when screened as part of the study protocol; 14 of which had a minimum of 1-year followup. Three of the 14 patients developed PTS compared to two out of 91 randomly matched patients without DVT [57].

### 5.2. Patient-Specific

It is unclear whether an association exists between increasing age, gender, and the development of PTS. Although two studies found a relationship between age and PTS, others did not [7, 13, 47, 48, 58]. Similarly, female gender has been inconsistently associated with PTS, while male gender was reported to be a weak risk [48, 59]. A correlation was confirmed, however, between PTS and obesity [60]. In a small cohort study of patients with DVT, those who developed PTS had significantly higher BMI than those who did not (29.6 versus 27.2 kg/m^2^, resp.; *P* = 0.022) [61]. This finding was confirmed by other studies and thus merits further evaluation of modifying obesity for PTS prevention and management [47, 58].

Although thrombophilic disorders predispose patients to developing first and recurrent DVT, the association to PTS has not been confirmed [62, 63]. Factor V Leiden or the prothrombin 20210A mutation were not associated with the development of PTS; in fact, one study showed a decreased risk when these factors were present [7, 48, 58, 60, 64].

Finally, inflammatory markers such as interleukin-6 and C-reactive protein are elevated in patients with DVT; however, an association between these markers and the development of PTS was not fully elucidated in a prospective study [62, 63, 65].

## 6. Risk Factors That Develop in the Long-Term Period Following the Management of DVT

### 6.1. Clinical Features

There is conclusive evidence that a recurrent ipsilateral DVT increases the risk of developing PTS by up to ten folds when compared with controls [2, 5, 7]. Further damage of valves and obstruction of blood flow due to recurrent DVT may be attributed to the increased incidence of PTS. In a study involving 316 patients with DVT, the risk for recurrent DVT was higher in patients with residual thrombus and led to increased vascular death resulting in vascular dysfunction [66]. Thus, preventing DVT in moderate-to-high-risk patients and providing adequate therapy for treatment are crucial to prevent recurrent DVT and consequently PTS.

Although residual thrombosis plays an important role in recurrent DVT, the association to PTS is not definitive. Two studies examined this issue; one found no association, while another reported an odds ratio of 1.69 (95% CI 1.23–2.32) [29]. Moreover, the role of residual thrombosis and popliteal valve reflux in the development of PTS was assessed in 180 patients diagnosed with acute proximal DVT followed for a minimum of 3 years. The relative risk was 1.6 (95% CI 1.0–2.4) in patients with residual vein thrombosis and 1.7 (95% CI 1.2–2.3) in patients with persistent venous obstruction alone or combined with popliteal valve reflux [29, 67]. In another study involving 93 patients with distal and proximal DVT followed up to 6 years, valve reflux had a predictive value while residual thrombosis had a weak-to-no association [29, 58]. These results were confirmed in another study which reported an association between elevated peak reflux velocity and the development of PTS [68].

Finally, a weak association between elevated D-Dimer levels after the withdrawal of anticoagulants and the development of PTS was reported (OR 1.9, 95% CI 1.0–3.9). Since D-Dimer has been implicated in predicting thrombotic recurrences, its role in the PTS should be further investigated [69, 70].

### 6.2. Patient-Specific

The quality of anticoagulation therapy when given to manage acute thrombosis constitutes a risk factor for the development of PTS. A study of patients treated with Vitamin K antagonists for a mean of 3 months (target INR 2.0-3.0) revealed an association in patients who had a subtherapeutic INR for more than 50% of the time and the development of PTS. In these patients, the risk was increased by about 3 folds and could be attributed to suboptimal clot resolution due to the poor quality of anticoagulation [47]. The intensity of anticoagulation was also examined in another study involving patients treated for unprovoked proximal DVT for an average of 2.2 years. Patients were randomized to a target INR of 2.0-3.0 or 1.5–1.9. The study did not find a difference in developing PTS between the two groups [58]. Finally, in a cohort study of 406 patients treated for a first DVT for a median of 60 months, there was no association between the duration of therapy (<6 months, 6–12 months, or >12 months) and the development of PTS [48].

## 7. Prevention of PTS

There are various measures to prevent PTS in patients diagnosed with acute DVT. These include preventing recurrent ipsilateral DVT, modifying risk factors such as obesity, treating venous obstruction via stenting and angioplasty, and surgically repairing or replacing venous valves [7, 20, 47, 58, 71, 72]. Moreover, catheter, directed thrombolysis has also shown to improve valvular reflux and vein patency and is recommended for selected patients diagnosed with iliofemoral DVT by practice guidelines [73–75].

Graduated elastic compression stockings can also be used in the management of PTS due to their effects on decreasing edema and venous hypertension, and improving tissue microcirculation [76]. The current guideline for the management of venous thromboembolism disease provides the highest recommendation for elastic compression stockings (Grade 1A) in those patients diagnosed with a DVT so, there is minimal patient risk and cost with high likelihood of preventing PTS [75]. This recommendation is based upon the results from several trials that support the use of compression stockings in the prevention of PTS. Brandjes et al. randomized 194 patients diagnosed with a first episode of proximal DVT to either daily use of sized-to-fit graded elastic compression stockings (40 mm Hg at the ankle, 36 mm Hg at the lower calf, and 21 mm Hg at the upper calf) for at least two years or no stockings (control group). Proximal DVT included thrombi involving the popliteal vein or above, irrespective of the presence of calf vein thrombi. All patients received treatment with heparin for at least 5 days and continued on Coumadin for 3 months. Mild-to-moderate PTS occurred in 20% of patients receiving stockings compared to 47% in the control group (*P* < 0.001), whereas severe PTS occurred in 11% of patients receiving stockings compared to 23% in the control group (*P* < 0.001). In both groups, most cases of PTS occurred within 24 months of the acute DVT event. There was no difference in recurrence of DVT between the two groups [38]. Similarly, in a study by Prandoni and colleagues, 180 patients with a first episode of proximal DVT were randomly assigned to below-knee compression stockings (30–40 mm Hg) for two years or no stockings (control group). The cumulative incidence of PTS in patients with stockings versus the control group was reduced after 6 months (21.1% versus 40%, resp.), 1-year (22.2% versus 46.7%, resp.), and 2 years (24.5% versus 49.1%, resp.) [7].

In contrast, in a 3-part study by Ginsberg and coauthors, 47 patients diagnosed with first episode of proximal DVT (involving the popliteal or more proximal vein) with evidence of venous valvular incompetence were randomized to either below-knee stockings (20–30 mm Hg) or matched placebo stockings. There was no statistical difference in the development of PTS between patients allocated to stockings versus placebo (0% versus 4.3%, resp.; *P* = 0.49) within a mean followup period of 57 months. However, the small number of patients enrolled in this study precludes definitive conclusions [40]. A study of acute proximal DVT, 53 patients were randomized to bed rest and no compression, boot bandages plus walking, or compression stockings plus walking, the authors reported a reduction in the incidence and severity of PTS in the mobile group with compression compared to the bed rest group (assessed by the Villalta-Prandoni scale; *P* < 0.01) [41]. These findings were consistent with another similarly designed trial conducted by the same primary author [39]. A more recent study by Ashwanden et al. randomized 169 patients with a first or recurrent proximal DVT to either ready-to wear flat-knitted below-knee stockings (26.3 to 36.1 mm Hg) or no stockings (control group). Proximal DVT included thrombi involving the popliteal vein or more proximal veins (femoral and iliac). All patients received heparin initially followed by an oral anticoagulation and compression stockings for at least 6 months. After finishing the 6 months of therapy, patients were randomized to continue treatment with either stockings or no stockings. The primary end point defined as the occurrence of emerging Postthrombotic skin changes according to a CEAP classification of 4 or greater developed in 13.1% of patients in the stocking group compared to 20.0% in the control group (hazard ratio [HR], 0.60; 95% CI, 0.28–1.28; *P* = 0.19) with a mean followup of 3.2 years and 2.9 years, respectively. Five additional patients in the control group requiring compression therapy for PTS were not included in the primary end point. Of note, women in the stocking group experienced a significant reduction in the development of severe skin changes (HR, 0.11; 95% CI, 0.02–0.91) compared to men (HR, 1.07; 95% CI, 0.42–2.73). This gender difference could be attributed to increased compliance as men were found to be 4 times less likely to wear the stockings compared to women (odds ration [OR], 4.1; CI, 1.0–16.0; *P* = 0.05) [42]. (Table 3) Individualized duration of elastic compression therapy to prevent PTS was also assessed in a study involving 125 patients with acute proximal DVT involving the popliteal, femoral, or common femoral veins. Results were consistent with findings from the trial by Ten Cate-Hoek et al. suggesting that patients with a low probability of developing the syndrome can be identified as early as 6 months after the thrombotic event [77].

Furthermore, a metaanalysis of the first three trials including a total of 421 patients supported the use of compression stockings (30–40 mm Hg) after DVT in reducing the risk of any PTS (odds ratio [OR] 0.3; 95% CI; 0.2–0.48) and of severe PTS (OR 0.39; 95% CI; 0.2–0.76) [78]. In a recent metaanalysis of 5 randomized trials including a total of 580 patients with DVT, mild-to-moderate PTS occurred in 22% of patients treated with compression stockings compared to 37% in the control group (relative risk = 0.52). Severe PTS and any PTS occurred in 5% and 26% of patients treated with compression stockings compared to 12% and 46% in the control group, respectively (relative risk = 0.38 and relative risk = 0.54, resp.) [79].

Although these data support using compression stockings to reduce the development of PTS, each previously mentioned trial had limitations that could affect the generalizability of the results. These include small sample size, single center recruitment, and bias due to lack of blinding and subjectivity of some elements of the scales used to diagnose PTS. The SOX Trial is a double-blind multicenter study that is currently under investigation to evaluate the effectiveness of compression stockings in the prevention of PTS. A total of 800 patients with proximal DVT will be randomly assigned to either compression stockings worn on the affected leg for 2 years or inactive stockings (placebo). The results of this trial should help answer questions regarding the use of compression stockings given the limitations of the previous trials [80].

## 8. Conclusion

PTS is a burdensome and costly syndrome that may affect up to 50% of patients diagnosed with DVT within the first two years. Studies have identified risk factors for developing this syndrome including obesity, older age, and poor quality of anticoagulation therapy. Several modalities have been used to prevent the development of PTS including compression stockings. The role of compression stockings including use and duration of use will be further clarified and confirmed with ongoing research. Limitations to using the compression stockings include compliance which is necessary to optimize effectiveness. It has been suggested that knee-length stockings are easier to apply and more comfortable than thigh-length stockings with similar physiologic outcomes [81]. Moreover, individualizing duration of treatment may also increase compliance.

## Figures and Tables

**Table 1:** Clinical Features of PTS.

Symptoms; worse with activity	Signs
Pain	Edema
Swelling	Hyperpigmentation
Heaviness	Venous eczema
Fatigue	Telangiactasias
Tingling	Secondary varicose veins
Itching	Lipodermatosclerosis
Cramps	Redness, cyanosis
Bursting sensation	Ulcers

**Table 2:** Clinical Scales for assessing the presence and severity of PTS.

PTS Scale	Criteria used to diagnose PTS	Comments
Ginsberg et al. [34]	Pain and swelling of limb > 1 month duration, typical character (worse at end of day or with prolonged sitting/standing, better after night's rest and leg elevation) that occurs ≥ 6 months after acute DVT AND Objective evidence of valvular incompetence (diagnosed via plethysmography or venous Doppler): (i) if both criteria are present, PTS is diagnosed, (ii) Global Rating Questionnaire used to rate overall improvement or worsening of PTS over time	Developed specifically for PTS; does not rate the severity of PTS

Villalta et al. [35]	5 Symptoms (pain, cramps, heaviness, pruritus, paresthesia) 6 signs (edema, skin induration, hyperpigmentation, ectasia, redness, pain during calf compression) Each rated as 0 (absent), 1 (mild), 2 (moderate) or 3 (Severe) Points are summed. A total score of 0–4: No PTS 5–14: Mild/Moderate PTS ≥15, or presence of ulcer: Severe PTS	Developed specifically for PTS; rates the severity of PTS

CEAP Classification* [36, 37]	Patients with chronic venous disease classified into 1 of 7 clinical classes (Class 0–6) according to the presence of clinical signs. Each class may include signs present in lower-order class. Class^†^: (0) Symptoms only; no visible or palpable signs of venous disease (1) Telangiectasias, reticular veins, malleolar flare (2) Varicose veins (3) Edema, no skin changes (4) Skin changes (e.g., pigmentation, eczema, lipodermatosclerosis) (5) Skin changes with healed ulcer (6) Skin changes with active ulcer Each clinical class is then subclassified as to: Etiology (congenital, primary, secondary) Anatomy (superficial, deep, perforator veins) Pathophysiology (reflux, obstruction, both)	Was developed for chronic venous disease in general and not for PTS; does not rate the severity of PTS

PTS = postthrombotic syndrome; *CEAP: Clinical-Etiology-Anatomic-Pathophysiologic. Modifications of CEAP (clinical severity score, venous segmental disease score) have been proposed.

^†^Increasing CEAP class is intended to reflect increased severity of signs of chronic venous disease; symptoms and their severity are not considered.

**Table 3:** Study Characteristics.

First author/year published	Reference	Number of patients per group (Stocking/Control)	Stocking characteristics	Stocking duration	Followup
Brandjes et al. 1997	[38]	96/98	Graduate compression stocking below knee (i) 40 mm Hg at ankle (ii) 36 mm Hg at lower calf (iii) 21 mm Hg at upper calf	≥2 years	≥5 years in both groups

Partsch and Blättler 2000	[39]	30/15	Inelastic compression bandages [Unna boots on the lower leg, adhesive bandages on the thigh] (*n* = 15) or Thigh-length compression stockings (*n* = 15)	9 days	None

Ginsberg et al. 2001	[40]	42/40	Elastic compression stocking below knee (*n* = 38) or thigh (*n* = 4) (i) 20–30 mm Hg	1.4–4.6 years	1.4–4.6 years in stocking group; 1.8–4.9 years in control group

Partsch et al. 2004	[41]	26/11	Thigh length elastic compression stocking (*n* = 13) or Gauze zinc oxide and calamine impregnated bandage on lower leg and firm thigh adhesive bandage (*n* = 13)	2 years	2 years in both groups

Prandoni et al. 2004	[7]	90/90	Graduate below-knee compression elastic stockings (i) 30 to 40 mm Hg at the ankle	2 years	3–5 years in both groups

Aschwanden et al. 2008	[42]	84/85	Compression stocking below knee (i) 26.3–36.1 mm Hg at ankle	3.2 years	3.2 years in the stocking group; 2.9 years in control group
